# A potential stormwater management intervention: Compost incorporation improves infiltration rate and associated soil hydro-physical properties

**DOI:** 10.1371/journal.pone.0355866

**Published:** 2026-08-14

**Authors:** Md Mahfuz Islam, Richard A. McLaughlin, Joshua L. Heitman

**Affiliations:** 1 Department of Crop and Soil Sciences, North Carolina State University, Raleigh, North Carolina, United States of America; 2 Department of Planning and Development Services, Watershed Management Division, Wake County Government, North Carolina, United States of America; Cranfield University, UNITED KINGDOM OF GREAT BRITAIN AND NORTHERN IRELAND

## Abstract

Urbanization associated construction operations, while necessary for infrastructure development, can result in highly disturbed and intentionally, or inadvertently, compacted soils. Compost incorporation into these disturbed soils is a relatively simple intervention that may alleviate compaction and increase important post-construction soil physical properties such as water infiltration capacity. Results in the literature about compost amendment effects on soil hydraulic properties are mixed, and compost experiments rarely test more than a single amendment rate or type concurrently, often without incorporation. To address this research gap, we conducted a laboratory study using soil columns filled with either woodchips-food waste (Compost 1) or poultry manure-food waste (Compost 2) compost mixed with silt loam or clay loam soils at 0, 15, 30, and 50% rates (by volume). Water (10 cm depth) was infiltrated into the columns at 15-day intervals over six months to assess the effects of compost incorporation on soil hydraulic properties. After six months, bulk and particle densities decreased, while porosity, field capacity, and plant available water increased at 30 and 50% compost rates. Magnitudes of effects differed by soil type, but compost type had no effect. Infiltration rates gradually stabilized after two months, possibly due to reduced compost decomposition and consolidation over time. Infiltration rates were highest for 50% compost, followed by 30% and 15%, and lowest for the 0% treatments. Infiltration rates were about 10, 5, and 1.5X that of the 0% compost (control) treatment, respectively. Results suggested that a minimum of 30% compost incorporation may be required for improving stormwater infiltration-related soil properties, and that peak infiltration rates observed after initial compost addition may decline, but compost amendment leads to sustained improvement in infiltration rates for at least several months after incorporation.

## Introduction

The United States Environmental Protection Agency has identified soil erosion and stormwater runoff as the major nonpoint sources of water pollution, especially in urban and suburban areas [1]. Urbanization, particularly the construction of structures such as roads and buildings, can lead to severely disturbed areas in which the soil is intentionally compacted to increase its strength or unintentionally compacted by heavy construction machinery [2–4]. Compaction makes both the surface and subsurface soils impervious, resulting in greater surface runoff volume [5–6] and bulk density [7–9] and reduced infiltration rate [2,4], soil structure [10], and porosity [11–12]. The increased runoff may carry sediment loads, toxic chemicals, and nutrients that reach nearby water bodies, seriously harming water quality [1]. For example, the Pennsylvania Department of Environmental Protection [13] estimated that about 18% of annual rainfall becomes surface runoff under natural conditions, while up to 95% of the rainfall transforms into surface runoff in developed areas.

Several post-construction soil management strategies have been devised for stormwater management systems and low-impact development practices to reduce runoff volume and pollutant loads, thereby safeguarding water quality and aquatic habitats through infiltration, evapotranspiration, filtration, storage, and detention [14–16]. The post-construction soil management strategies used to improve stormwater infiltration include organic amendments (e.g., compost, biosolids, biochar), soil decompaction techniques (e.g., deep tillage), mulching, topsoil management [2,17–19], and engineered soil media [20]. These strategies are commonly incorporated into stormwater management practices such as bioretention systems, bioswales, and permeable pavements to enhance infiltration, reduce runoff, and improve pollutant removal [21–23]. Among them, the application of compost amendment has attracted widespread interest in heavily compacted post-construction sites, especially where high stormwater infiltration capacity is mandated by regulation [24]. Growing evidence suggests that compost incorporation into post-construction urban soils can help restore the natural function of soils through increasing soil hydraulic conductivity, porosity, and aggregate stability and reducing bulk density [6,7,19,25]. However, detailed research on compost application in post-construction soils is still rare. Rivier et al. [10] described that six hundred research articles on compost usage were published until 2020, but only 10% addressed soil hydraulic properties and soil structure. The majority of studies only address surface compost applications or shallow incorporation (typically within the upper 5–15 cm of the soil profile) [26–28], although incorporating compost at deeper layers (approximately 30 cm or greater) has the potential to alleviate subsurface soil compaction and thus could greatly enhance stormwater infiltration [2,29,30]. Deep compost incorporation is typically achieved during site preparation through deep tillage (subsoiling) followed by mechanical mixing of compost into the loosened soil profile using excavation equipment, rotary tillers, or soil blending equipment [2,7,25,31].

Appropriate compost rates and compost types for specific degraded soil textures have not been established experimentally for increasing infiltration rates. In addition, most compost incorporation studies have only evaluated a single compost rate and single compost types, namely yardwaste-based [2,32–34], sludge-based [35], or cattle manure-based composts [36]. Different studies separately reported that soil texture, compost types, and compost rates can greatly affect the function of compost [17,32,37–39], suggesting that these factors must be considered while selecting compost rates for application in construction site soils. Barzegar et al. [40] examined the impact of three distinct compost types and reported improvement in infiltration rate, aggregate stability, water retention, and bulk density after one year, but compost type had no effect. Kranz et al. [17] studied the effects of five compost rates on saturated hydraulic conductivity and water retention on three soil types. They intentionally maintained consistent porosity levels across soil-compost blends, as compost addition may alter soil bulk density and material mean particle density, affecting porosity. This approach (i.e., controlling porosity) enabled them to make a direct comparison of soil hydraulic properties with varying rates of compost that were attributable to material composition and particle size distribution, but independent of concurrent changes in porosity associated with compost application. However, this approach also limited examination of the effects of increase in porosity often accompanying compost application, which also has an important practical effect on soil hydraulic properties [25]. Excluding the effect of porosity changes accompanying compost application also precludes examining the subsequent changes in soil hydraulic properties that occur over time with the settling of amended soil under repeated wetting and drying, and compost breakdown.

Most previous studies have evaluated compost amendments under field conditions, where hydrologic responses are influenced by rainfall variability, vegetation establishment, traffic, and maintenance practices [2,7,25,31]. Controlled laboratory experiments minimize these confounding factors and allow the independent effects of compost incorporation rates, compost types, and soil texture on soil physical properties and infiltration to be quantified. Such studies provide mechanistic understanding that complement, rather than replaces, field-scale investigations. Although laboratory conditions cannot fully replicate field environments, they provide controlled conditions for evaluating treatment effects while minimizing variability associated with rainfall intensity, antecedent moisture, vegetation dynamics, machinery traffic, and spatial heterogeneity. Therefore, the observed improvements should be interpreted as demonstrating the potential effectiveness of compost incorporation under controlled conditions rather than exact predictions of field performance.

As urban and peri-urban areas continue to expand, soil compaction associated with development is becoming increasingly widespread, leading to increased stormwater runoff and water pollution [1,41–43]. To address these challenges, simple, effective, and widely applicable stormwater management strategies are needed to restore soil function and improve infiltration. This study tested the effects of two types of compost, each applied at four rates to two soils, on soil infiltration rate, bulk density, particle density, porosity, and plant available water storage capacity. While infiltration capacity increases are a primary goal with compost amendment in stormwater management, the capacity of the amended soils to maintain vegetation between infiltration events by storing plant available water is also an important consideration to support long-term soil function with low inputs (i.e., rain-fed systems). Amended soils were subjected to a series of infiltration events over a period of six months. We hypothesized that soil-incorporated compost would increase water infiltration and water storage with variations between compost types, but that this effect would diminish over time with subsequent infiltration events. Additionally, we hypothesized that there would be further enhancements to soil hydro-physical properties as the rate of compost application increased. Our results are intended to help aid design of simple stormwater infiltration improvement practices for soils disturbed by development.

## Materials and methods

### Soil and compost sources

We tested two types of compost that were produced at the North Carolina State University Compost Facility and Research Cooperative (Raleigh, NC, USA). The first compost (relatively coarse) was a mixture of 50% wood chips and 50% food waste (hereafter Compost 1), whereas the second compost (relatively fine) was composed of 50% poultry manure and 50% food waste (hereafter Compost 2) (Table 1). The silt loam and clay loam soils (identified hereafter according to these USDA textural classifications) we used were local subsoil materials supplied by local landscape vendors and were representative of construction site fill material (Table 1). The collected soils and composts were air-dried for two weeks and soil was sieved through a 2 mm stainless steel sieve. The particle size analysis of soil was conducted using the hydrometer method [44], and the particle size distribution of compost was measured using the American Geophysical Union’s (AGU) classification system of [45]. In the AGU system, larger compost particles (2–9.5 mm) are defined as fine gravel sizes and smaller particles (≤ 2 mm) are coarse sand to clay sizes. Brookside Laboratories, Inc. (New Breman, OH, USA) conducted initial organic carbon and total nitrogen analysis of soils and compost materials (Table 1).

**Table 1:** Carbon, nitrogen, and particle size distribution of the studied soils and composts.

	Soil			Compost	
Property	Silt Loam	Clay Loam	Property	Compost 1	Compost 2
***Chemical Composition***					
**Organic C (%)**	0.95	2.01	**Organic C (%)**	43	28
**Total N (%)**	0.03	0.05	**Total N (%)**	2.3	1.4
**C/N ratio**	32	40	**C/N ratio**	19	20
***Particle size distribution***					
**Sand (2–0.05 mm, %)**	26	21	**>2–9.5 mm (%)**	70	50
**Silt (<50−2 µm, %)**	62	41	**≤2 mm (%)**	30	50
**Clay (<2 µm, %)**	12	38			

### Column preparation

**Fig 1:**
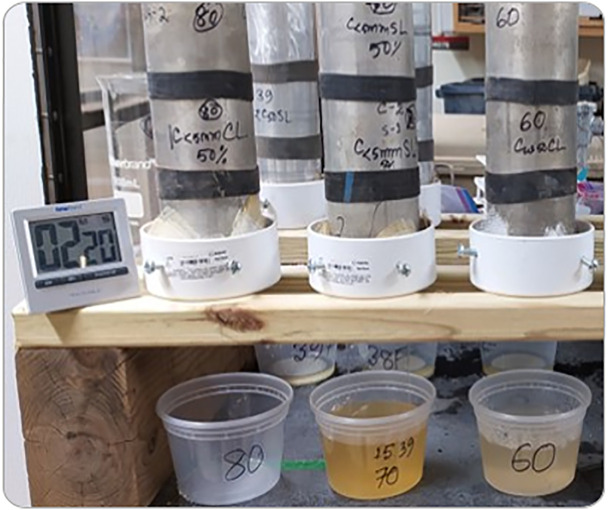
Soil-compost packed infiltration columns.

Columns (total length 15.2 cm) were prepared by connecting two aluminum rings, each 7.6 cm in height and diameter, and attaching a drilled PVC end cap at the bottom. About 0.5 cm layer of woven iron mesh and polyester cheesecloth was placed at the bottom of the column to prevent clogging the holes in the caps and loss of soil and compost particles (Fig 1).

Each type of compost was mixed with two soil types (silt loam or clay loam) at 0, 15, 30, and 50% rates on a volume basis. Homogenous mixtures of respective soil and compost rates were prepared by hand mixing on a clean plastic sheet. Then, the columns were packed with the homogenous mixture of test samples and compacted with a bulk density core hammer (1 kg), freely falling from 10 cm height 10 times to eliminate void spaces in the column. Additional material was added until packing resulted in the complete filling of the column to the surface (15 cm depth). An additional aluminum ring (7.6 cm height) was attached to the top of the filled soil column with rubber tubes for holding water during infiltration tests. Each treatment was replicated three times (2 soils × 2 composts × 4 rates × 3 reps = 48 columns), and columns were placed randomly on a wooden frame for the water infiltration study. Columns were maintained in the laboratory at approximately 22 °C for the duration of the experiments. The initial bulk densities of all the columns were calculated from the known dry mass of the soil-compost mixtures and the volume of the columns. We also measured the bulk densities of unmixed Compost 1 and Compost 2 (0.27 and 0.31 g cm^-3^, respectively) and silt loam and clay loam soils **(**1.42 and 1.24 g cm^-3^, respectively) following the same filling procedure.

### Studied parameters

#### Infiltration rate.

A water depth equivalent of 10 cm (~65% of the pore volume) was passed through the columns at 15-day intervals for 6 consecutive months to determine infiltration rates. A piece of cheesecloth was used at the top of the soil column to limit disturbance during water additions. The columns were pre-wet with a 2-cm water depth 3-h prior to the infiltration measurements each time. For infiltration rate, time was recorded from the initial addition of water until 1 cm remained ponded on the soil surface (i.e., 9 cm of water had infiltrated) [46,47]. In contrast to the modified ASTM C1701 method, where the end time is determined when there is no free water on the surface, we calculated the end time by observing the presence of the last 1 cm of water on the surface, aiming for better timing precision. The infiltration rate was calculated as the infiltration depth (9 cm) divided by the time over which this infiltration occurred.

After completion of the experiment (i.e., 6 months after initiation), we separated the empty ring (water ponding column section) from the rest of the column and recorded the loss in height of the soil within the remaining column (compared to the original packing height). The corrected soil volume within the column was calculated by subtracting the loss in height from the original column height of 15.2 cm. Then, soil-compost mixtures were used to determine water retention, bulk density, particle density, and porosity.

#### Bulk density, particle density, and porosity.

Soil samples were oven-dried at 105°C until a constant weight was recorded (~48 h). Bulk density was calculated at the ratio of the dry mass to volume, where volume was corrected for measured loss in sample height at the end of the experiment. The particle density of the samples was determined by the gas pycnometer method [48,49]. The 105°C oven-dried samples were placed in a desiccator chamber and kept overnight. The next day, dry mass was recorded and samples were placed in the gas pycnometer (AccuPyc II 1340, Micromeritics, USA). Inert helium gas was used as the displacement medium for the gas pycnometer that measured the soil solid volume of a single sample 10 times, including the final mean and standard deviation. Particle density was computed as the ratio of dry soil mass to its volume as determined by the gas pycnometer. Bulk density and particle density ratios were used to calculate the porosities of the treatments by subtracting the ratios from 1.

#### Water retention.

Samples were taken at the end of the study (6 months) for the determination of water retention at field capacity (33 kPa) and wilting point (1500 kPa) using high-range pressure plate extraction [50]. About 100 g of each sample was placed in a rubber ring on a pressure plate and saturated for 2 days, due to the high organic matter content of the samples. Next, samples were placed inside a pressure chamber and equilibrated for 10 days at the specified pressures. Samples were dried at 105°C for 48 hours and re-weighed in order to calculate water content. Volumetric water contents at field capacity and wilting point were calculated by multiplying the water content with final bulk densities measured from the columns. Finally, plant available water was estimated by subtracting wilting point water content from field capacity water content.

### Statistical analysis

R version 4.2.1 [51] was used for three-way analysis of variance (ANOVA) to evaluate differences and relationships between soil types, compost types, and compost rates regarding bulk density, particle density, porosity, and water retention after 6 months of study. Since infiltration rate measurements were run at 15-days intervals 13 times over six months (0–6 months) on the same set of columns, a repeated measure ANOVA was used to assess the effects of the time factor on infiltration rate. Repeated measure ANOVA was conducted in IBM SPSS (Version 29, NY, USA), where the default compound symmetry covariance structure was used for fitting the data. The Greenhouse–Geisser method was used to assess within-subject effects in repeated-measures ANOVA when Mauchly’s Test of Sphericity was significant at the p < 0.05 level. A simple linear regression was run for compost rates and studied soil properties. Significant interactions identified in ANOVA were further assessed using Tukey’s Honestly Significant Difference (HSD) test (p = 0.05).

## Results and discussion

### Bulk density, particle density, and porosity

The results of the three-way ANOVA analysis highlighted the significant main effects of soil types and compost rate on bulk density and porosity and only compost rate on particle density (S1 Table). The interactive effects of soil type-compost type and soil type-compost rate for bulk density and porosity indicated that the impact of compost types might depend on their application rate in different soil textures. However, particle density only substantially depended on compost rates. These interactions implied that soil texture, compost rate, and compost type collectively influenced the shaping of these three interdependent soil properties.

**Fig 2:**
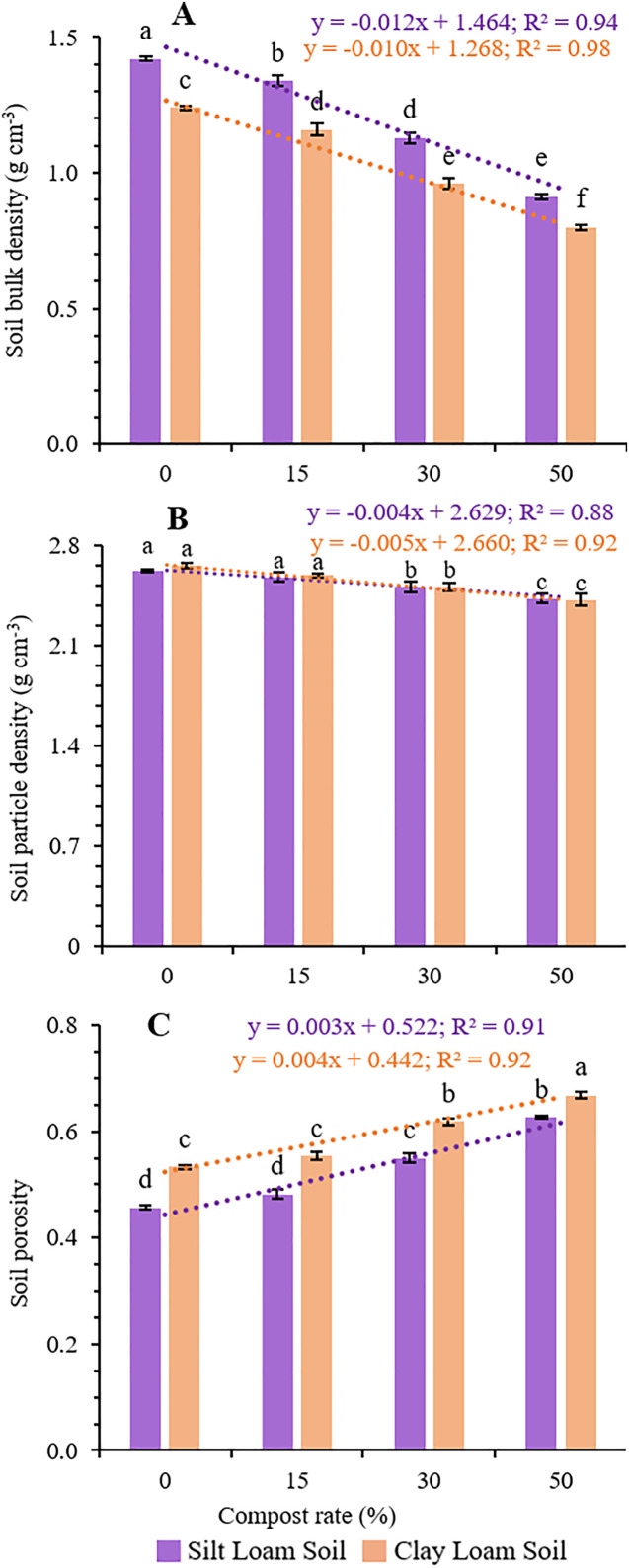
Effects of compost incorporation rates on A) bulk density, B) particle density, and C) porosity in silt loam and clay loam soils after 6 months. Bars represent standard errors. Letters indicate significant interactions among soil type and compost rate (Tukey’s HSD; p ≤ 0.05).

The relationship between compost rate and bulk density (R^2^ = 0.94 and 0.98) and particle density (R^2^ = 0.89 and 0.92) was negative and linear in silt loam and clay loam soils, but for porosity (R^2^ = 0.91 and 0.92) the relationship was positive and linear (Fig 2). Overall, bulk density and particle densities were decreased, and porosity was increased with each increment in compost rate except 15% addition in silt loam and clay loam soils, compared to the 0% compost treatments. Although bulk density was reduced at all rates for both soils, porosity did not increase until 30% compost addition. We observed a substantial increase in the porosities at the 30 and 50% compost rates that varied between 20–40% increases in porosity in silt loam and 14–27% increases in porosity in clay loam soils (Fig 2C). The increased porosities, and reduced bulk and particle densities of the mixtures are due directly to the low densities and large particle sizes (Table 1) of the compost amendments.

Similar reductions in bulk density have been reported following compost incorporation into urban soils, although the magnitude of improvement has varied with soil texture, compost application rate, and incorporation depth. Kranz et al. [7] found no change in bulk densities with tilling 10% compost in the upper 15 cm of sandy loam soil. Likewise, Castellini et al. [52] reported only small changes in bulk density and porosity in clay soils amended with 75 kg m^-2^ compost in 30 cm deep soil boxes. Rivier et al. [10] studied the changes in porosity with five types of compost addition, and they reported an increase in macro porosity in sandy soil between 1.1–4.98%. In contrast, the present study demonstrated substantially greater improvements in soil physical properties, with the 30% compost treatment increasing porosity by approximately 20% in silt loam and 17% in clay loam soils. These findings suggest that higher compost incorporation rates can produce more pronounced improvements in soil structure and pore development, particularly in compacted fine-textured soils, thereby enhancing their potential for stormwater infiltration.

### Infiltration rate

Soil type and compost rate significantly affected the infiltration rate in all studied events, while compost type affected no event (S1 Table). There were interactions of compost type with soil type and compost rates for a few events. However, repeated measure ANOVA showed that the main effects of soil type, compost rate, and time emerged as highly influential factors (S1 Table). The interactions of soil × compost rate, soil × time, compost rate × time, and soil × compost rate × time underscored the linked nature of these factors and emphasized the importance of considering each of these factors to explain stormwater infiltration into soil.

**Fig 3:**
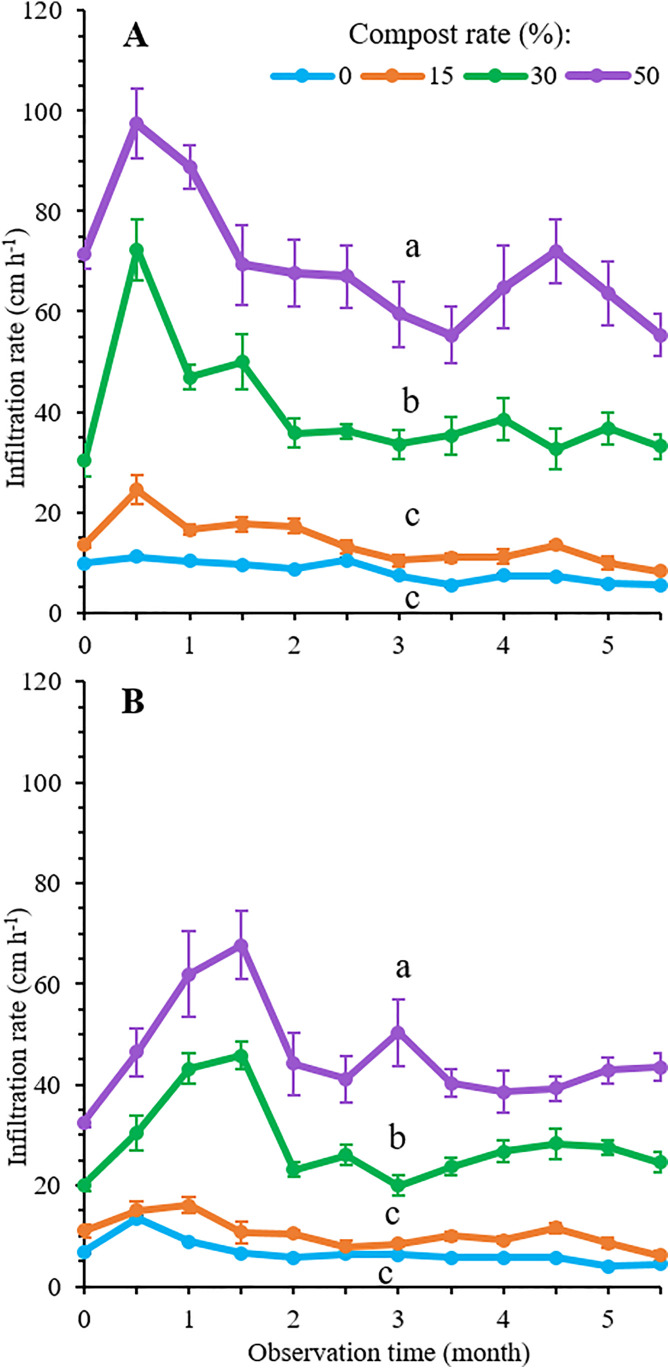
Infiltration rates at different infiltration events in A) silt loam and B) clay loam soils. Bars represent standard errors. Letters indicate significant interactions among compost rates within the same observation time (Tukey’s HSD; p ≤ 0.05).

At every infiltration event, infiltration rates were highest for 50% compost, intermediate for 30%, lower for 15%, and lowest for control (0% compost) treatments in both soils (Fig 3). The maximum infiltration rates were observed at 0.5-month and 1.5-month events in silt loam and clay loam soils, respectively. Thereafter, the infiltration rates decreased for up to 2 months and finally became nearly stable for each compost rate. Compared to the 0% control, the mean infiltration rates were higher by approximately 1.5, 6, and 10X after 6 months of the experiment in both soils for 15, 30, and 50% compost rates, respectively. However, the magnitude of the mean infiltration rate value at all compost rates was higher in silt loam soil than in clay loam. Compost decomposition in the initial days after incorporation might cause void spaces inside the columns that increase infiltration in the initial events and, after that stabilize, possibly due to soil consolidation and reduced compost mineralization. A possible reason for the lowest infiltration rate in the first event (0 month) could be the development of hydrophobic characteristics due to the drying of soil (gravimetric moisture content 3.38%) and compost (gravimetric moisture content 3.63%) before column preparation that slowed infiltration during the first few wettings, similar to initial wetting effects observed in organic substrates [e.g., 53].

Infiltration rates in silt loam and clay loam soils increased significantly at the 30% and 50% compost rates in all the events compared to 0% treatments. These results suggest that a minimum of 30% compost amendment rate may be required to substantially increase infiltration rates. This threshold is consistent with the corresponding increase in soil porosity observed at the 30% compost rate, indicating that improvements in pore structure likely contributed to the enhanced infiltration. Previous studies have also reported increased infiltration following compost incorporation across a range of soil textures [2,26,29,34,54], although some studies observed little or no improvement [30]. Kranz et al. [17] reported that a 50% compost addition increased saturated hydraulic conductivity by only 36.5% in silt loam soil. The relatively modest improvement, despite the high compost application rate, was likely due to the controlled porosity (0.5 m³ m ⁻ ³) and immediate measurement of properties, which likely limited the potential for additional pore development. In contrast, the present study observed much greater increases in infiltration, remaining evident after six months of repeated infiltration events, suggesting that compost-induced changes in soil structure under compacted conditions can have a substantial influence on hydraulic performance.

### Water retention

Both soil type and compost rate had effects on field capacity (FC) and wilting point (WP) water content (S1 Table), while only compost rate impacted plant available water (PAW). However, compost type did not have effects on these soil properties. The interactive effects of soil type and compost rate were observed for all three properties. These findings demonstrated that compost rates may influence plant available water, particularly in soils with silt loam and clay loam textures.

**Fig 4:**
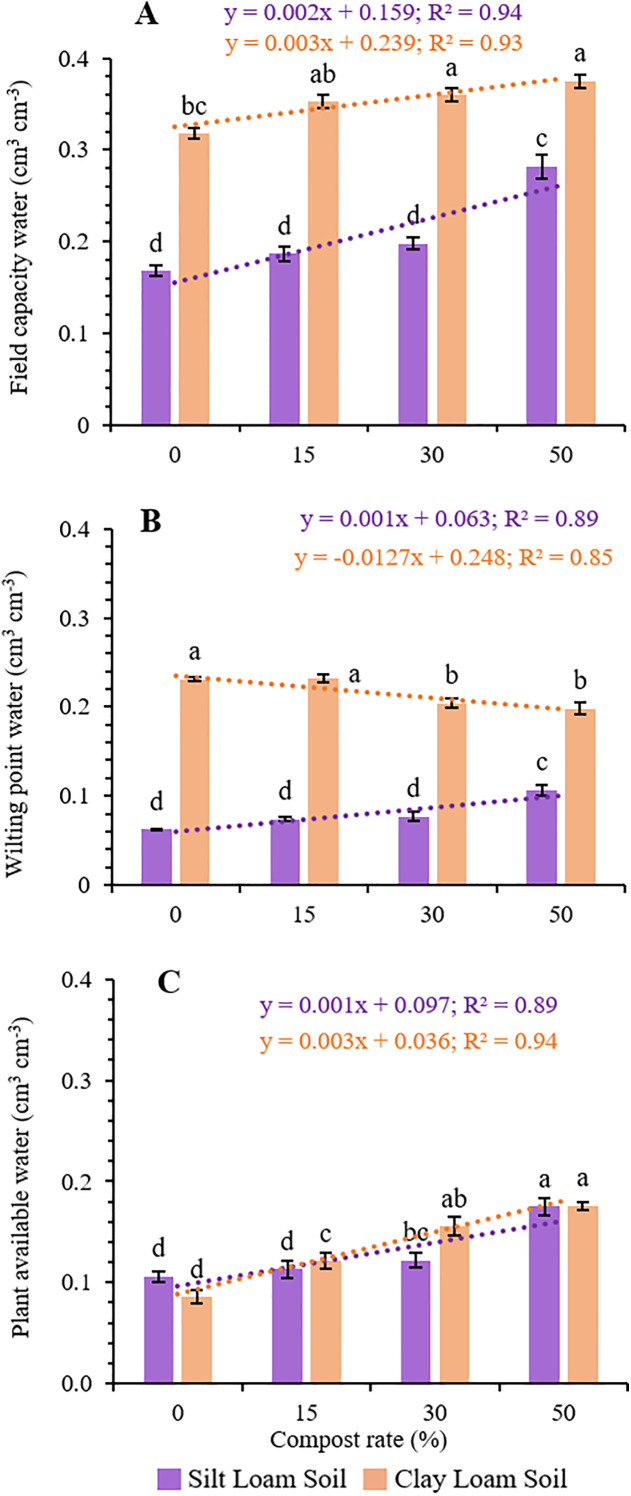
Effects of compost application rates on A) field capacity, B) wilting point, and C) plant available water content in silt loam and clay loam soils after 6 months. Bars represent standard errors. Letters indicate significant interactions among soil type and compost rate (Tukey’s HSD; p ≤ 0.05).

The relationship between compost rate and FC (R^2^ = 0.93 and 0.94), PAW (R^2^ = 0.94 and 0.89), and WP (R^2^ = 0.89 and 0.43) were positive and linear in silt loam and clay loam soils except for WP in clay loam soil (Fig 4). Overall, the silt loam and clay loam soils exhibited positive trends in FC and PAW contents with increasing compost rates. In silt loam soil, incorporation of compost led to an increase in FC, with a significant enhancement ranging from 65–70% at 50% compost rate, compared to 0% control. Clay loam soil showed a small significant increase beginning at 30% compost rate, and the response at 50% compost rate was also relatively small, with an increase of 16–20% compared to control. Contrasting trends were observed in WP between silt loam and clay loam soils. Silt loam soil exhibited a positive trend in WP, while clay loam soil displayed a negative trend with higher compost rates. The difference in pattern is attributable to particle-size distribution. The unamended clay loam, with approximately 3X the clay content as the silt loam, had large WP that was reduced with coarse particle additions from compost. Alternately, the unamended silt loam had relatively low WP that was increased by the addition of compost fines at the highest rate of addition. Consequently, the increases in PAW at 15, 30, and 50% compost treatments were more pronounced in clay loam soil (35–47, 70–93, and 96–114%) than in silt loam soil (3–10, 13–20, and 61–70%), despite comparable positive trends in FC with compost addition.

Our findings are consistent with several studies that reported compost amendments increase FC and PAW. For example, Weber et al. [55], Taban and Naeini [56], and Curtis and Claassen [33] documented similar positive effects of compost on soil moisture properties while Cogger [6] noted that compost-amended soils provide a more consistent water supply for plant growth. Similarly, Logsdon et al. [26] and Sax et al. [19] observed increased PAW in compost-amended soils. The present study extends these findings by demonstrating that the magnitude of improvement depends strongly on soil texture. Although FC increased in both soils, the greatest increases in PAW occurred in the clay loam soil because compost reduced wilting point while simultaneously increasing field capacity. In contrast, the silt loam soil exhibited increases in both FC and WP, resulting in comparatively smaller gains in PAW. These contrasting responses highlight the importance of considering soil texture when selecting compost application rates for stormwater management and vegetation establishment. The contrasting responses observed here are also consistent with Curtis and Claassen [32], who reported that compost effects on PAW varied among soil types, with decreases in sandy loam but increases in loam soils. Together, these findings suggest that the benefits of compost amendment for water storage are soil-dependent and are greatest where compost simultaneously enhances water retention while minimizing the amount of water held too tightly for plant uptake.

## Conclusion

This study aimed to determine the effects of compost incorporation, especially as affected by soil type, compost type, compost rate, and time, on stormwater infiltration-related soil hydro-physical properties. Compost application reduced bulk and particle densities and increased porosities in both silt loam and clay loam soils at the end of the six-month experiment. Although peak infiltration rates in compost-amended soils were observed after the first few infiltration events and subsequently declined, they stabilized over time. Infiltration rates were significantly higher at 30% and 50% (6 and 10X, respectively) compost rates, compared to controls, indicating a minimum of 30% compost amendment is likely necessary for substantial improvement of the infiltration rate. High compost rates (30 and 50%) increased field capacity and plant available water contents in both soils. These improvements are attributed to increased porosities due to compost incorporation. However, compost amendments also increased wilting point water content in silt loam soil but decreased it in clay loam soil, likely due to differences in clay contents of the unamended soils. This effect also contributed to differences observed in plant available water contents. Overall, a 30–50% compost incorporation demonstrated potential as a soil management approach for improving stormwater infiltration and other hydro-physical properties of disturbed urban soils. Compost type did not significantly affect the measured soil properties for the two compost sources evaluated in this study, suggesting that locally available or cost-effective compost sources may potentially be suitable for improving soil physical conditions; however, further research using a wider range of compost materials is needed to determine whether compost characteristics, such as feedstock source, maturity, and chemical composition influence long-term soil performance. Furthermore, this study was conducted under controlled mesocosm conditions, which may not fully represent field environments where factors such as soil heterogeneity, compaction variability, soil layering, vegetation establishment, rainfall patterns, and construction-related disturbances can influence infiltration behavior. Field-scale studies are needed to validate the effectiveness of compost incorporation as a stormwater management practice and to evaluate long-term performance, practical application methods, and optimal amendment rates under diverse urban soil conditions. Overall, compost incorporation appears promising as a stormwater management intervention for disturbed urban soils.

## Supporting information

S1 Table ANOVA Results for soil physical properties and infiltration. (PDF)

S1 File Raw data for analysis. (XLSX)
