# Supplementary material for: A potential stormwater management intervention: Compost incorporation improves infiltration rate and associated soil hydro-physical properties
Source: PLoS One. 2026 Aug 14;21(8):e0355866. doi: 10.1371/journal.pone.0355866 (PMC13475814; doi:10.1371/journal.pone.0355866)
Supplement: Supplementary file 1 [file pone.0355866.s001.pdf]

**Three-way ANOVA with F-values for soil physical properties after 6 months.**

| Factor | df | Bulk density | Particle density | Porosity | Field capacity | Wilting point | Plant available water |
|---|---|---|---|---|---|---|---|
| Soil | 1 | 266.707 *** | 1.300 | 244.114 *** | 576.603 *** | 1519.16 *** | 1.282 |
| Compost | 1 | 0.525 | 1.791 | 0.045 | 2.608 | 0.041 | 2.681 |
| Rate | 3 | 484.380 *** | 127.380 *** | 280.197 *** | 36.169 *** | 3.764 * | 38.767 *** |
| Soil × Compost | 1 | 5.500 * | 0.061 | 4.880 * | 0.033 | 0.194 | 0.240 |
| Soil × Rate | 3 | 3.716 * | 1.601 | 3.545 * | 8.241 *** | 24.397 *** | 4.123 *** |
| Compost × Rate | 3 | 0.225 | 0.717 | 0.399 | 0.553 | 0.049 | 0.434 |
| Soil × Compost × Rate | 3 | 1.427 | 0.533 | 0.927 | 0.067 | 0.428 | 0.230 |

*, **, and *** are significant at 0.05, 0.01, and 0.001 levels. df is degree of freedom.

**Monthly three-way ANOVA with F-values for infiltration rates from 0-6-month.**

| Factor | df | Time (month) | | | | | | | | | | | |
|---|---|---|---|---|---|---|---|---|---|---|---|---|---|
| | | 0 | 0.5 | 1 | 1.5 | 2 | 2.5 | 3 | 3.5 | 4 | 4.5 | 5 | 6 |
| Soil | 1 | 222.081 *** | 93.038 *** | 9.793 ** | 1.665 | 23.893 *** | 35.013 *** | 5.807 * | 14.329 *** | 14.705 *** | 21.253 *** | 16.970 *** | 13.008 ** |
| Compost | 1 | 3.538 | 0.043 | 0.008 | 0.645 | 0.014 | 0.089 | 0.688 | 0.037 | 0.385 | 3.092 | 0.065 | 1.009 |
| Rate | 3 | 464.398 *** | 115.883 *** | 135.269 *** | 89.788 *** | 85.241 *** | 124.741 *** | 69.666 *** | 115.083 *** | 60.633 *** | 101.595 *** | 124.124 *** | 165.704 *** |
| Soil × Compost | 1 | 1.311 | 8.105 ** | 1.093 | 3.436 | 4.259 * | 4.237 * | 0.491 | 0.000 | 0.010 | 0.008 | 0.325 | 0.022 |
| Soil × Rate | 3 | 87.789 *** | 24.356 *** | 5.913 ** | 0.140 | 3.665 * | 6.965 *** | 1.249 | 4.457 * | 4.544 ** | 11.896 *** | 5.027 ** | 2.547 |
| Compost × Rate | 3 | 5.951 ** | 0.516 | 1.276 | 0.753 | 1.232 | 0.664 | 0.256 | 0.479 | 0.264 | 0.947 | 1.878 | 0.351 |
| Soil × Compost × Rate | 3 | 3.246 * | 2.185 | 1.712 | 1.270 | 1.072 | 3.016 * | 0.553 | 3.838 * | 1.435 | 0.272 | 0.459 | 0.008 |

*, **, and *** are significant at 0.05, 0.01, and 0.001 levels. df is degree of freedom.

**Repeated Measure ANOVA for infiltration rates from 0-6-month.**

| Factor | df | Infiltration rate (cm $h^{-1}$) |
|---|---|---|
| **Between Subject Effects** | | |
| Soil | 1 | 101.841*** |
| Compost | 1 | 0.972 |
| Rate | 3 | 528.373*** |
| Soil × Compost | 1 | 0.213 |
| Soil × Rate | 3 | 25.147*** |
| Compost × Rate | 3 | 0.604 |
| Soil × Compost × Rate | 3 | 0.342 |
| **Within-Subject Effects** | | |
| Time | 4.294, 137.406 | 27.362**** |
| Time × Soil | 4.294, 137.406 | 6.565*** |
| Time × Compost | 4.294, 137.406 | 0.473 |
| Time × Rate | 12.882, 137.406 | 3.959*** |
| Time × Soil × Compost | 4.294, 137.406 | 2.729* |
| Time × Soil × Rate | 12.882, 137.406 | 3.260*** |
| Time × Compost × Rate | 12.882, 137.406 | 0.890 |
| Time × Soil × Compost × Rate | 12.882, 137.406 | 1.679 |

* and *** are significant at 0.05 and 0.001 levels. df is degree of freedom. Greenhouse–Geisser corrected degrees of freedom are used for the within-subject effects, following a significant Mauchly's Test of Sphericity at the $p < 0.001$ level.
